# (2*E*)-3-(4-Cyano­phen­yl)-1-(4,4′′-difluoro-5′-meth­oxy-1,1′:3′,1′′-terphenyl-4′-yl)prop-2-en-1-one

**DOI:** 10.1107/S1600536812023124

**Published:** 2012-05-26

**Authors:** Hoong-Kun Fun, Wan-Sin Loh, S. Samshuddin, B. Narayana, B. K. Sarojini

**Affiliations:** a X-ray Crystallography Unit, School of Physics, Universiti Sains Malaysia, 11800 USM, Penang, Malaysia; b Department of Studies in Chemistry, Mangalore University, Mangalagangotri 574 199, India; c Department of Chemistry, P.A. College of Engineering, Nadupadavu, Mangalore 574 153, India

## Abstract

In the title compound, C_29_H_19_F_2_NO_2_, the central benzene ring forms a dihedral angle of 56.92 (12)° with the cyano­benzene ring and dihedral angles of 40.91 (12) and 44.76 (12)° with the two fluoro­benzene rings. In the crystal, C—H⋯O and C—H⋯F hydrogen bonds link the mol­ecules into sheets lying parallel to the *ab* plane. The crystal packing also features C—H⋯π inter­actions involving the central benzene ring.

## Related literature

For background to terphenyls, see: Fun, Hemamalini *et al.* (2011 ▶); Fun, Shahani *et al.* (2011 ▶); Fun *et al.* (2012 ▶); Betz *et al.* (2011 ▶). For a related structure, see: Fun, Chia *et al.* (2011 ▶). For the stability of the temperature controller used in the data collection, see: Cosier & Glazer (1986 ▶).
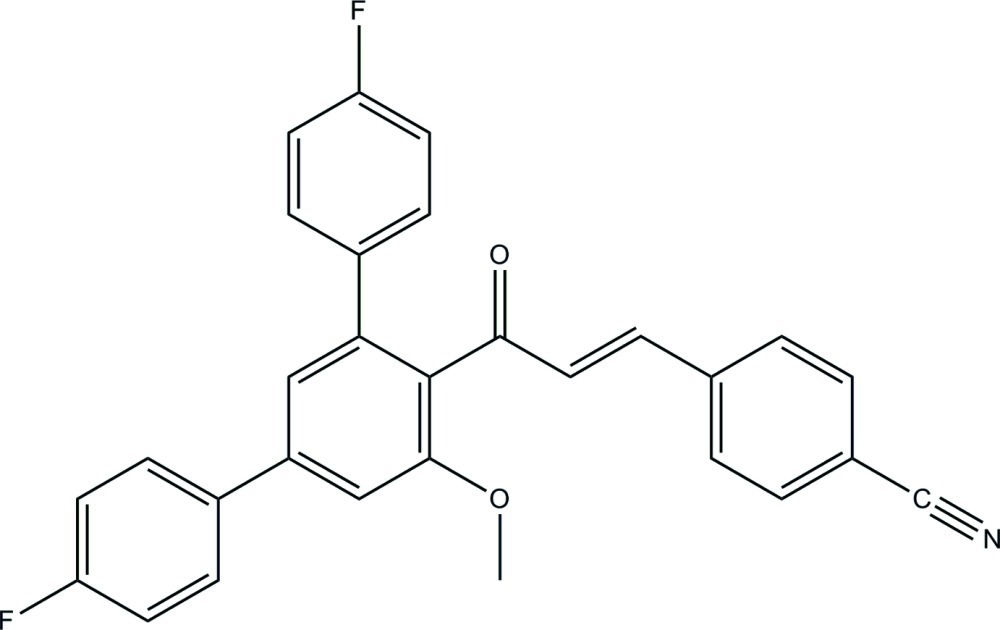


## Experimental

### 

#### Crystal data

C_29_H_19_F_2_NO_2_
*M* *_r_* = 451.45
Triclinic, 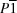

*a* = 6.9656 (2) Å
*b* = 11.2404 (3) Å
*c* = 14.6014 (3) Å
α = 96.108 (1)°
β = 90.415 (1)°
γ = 104.764 (1)°
*V* = 1098.51 (5) Å^3^
*Z* = 2
Mo *K*α radiation
μ = 0.10 mm^−1^
*T* = 100 K
0.34 × 0.20 × 0.12 mm

#### Data collection

Bruker APEXII CCD diffractometer
Absorption correction: multi-scan (*SADABS*; Bruker, 2009 ▶) *T*_min_ = 0.968, *T*_max_ = 0.989
17054 measured reflections
3828 independent reflections
2982 reflections with *I* > 2σ(*I*)
*R*_int_ = 0.036

#### Refinement

*R*[*F* ^2^ > 2σ(*F* ^2^)] = 0.050
*wR*(*F* ^2^) = 0.112
*S* = 1.03
3828 reflections
308 parameters
H-atom parameters constrained
Δρ_max_ = 0.31 e Å^−3^
Δρ_min_ = −0.23 e Å^−3^

### 

Data collection: *APEX2* (Bruker, 2009 ▶); cell refinement: *SAINT* (Bruker, 2009 ▶); data reduction: *SAINT*; program(s) used to solve structure: *SHELXTL* (Sheldrick, 2008 ▶); program(s) used to refine structure: *SHELXTL*; molecular graphics: *SHELXTL*; software used to prepare material for publication: *SHELXTL* and *PLATON* (Spek, 2009 ▶).

## Supplementary Material

Crystal structure: contains datablock(s) global, I. DOI: 10.1107/S1600536812023124/hb6802sup1.cif

Structure factors: contains datablock(s) I. DOI: 10.1107/S1600536812023124/hb6802Isup2.hkl

Supplementary material file. DOI: 10.1107/S1600536812023124/hb6802Isup3.cml

Additional supplementary materials:  crystallographic information; 3D view; checkCIF report

## Figures and Tables

**Table 1:** Hydrogen-bond geometry (Å, °) *Cg*1 is the centroid of the C13–C18 ring.

*D*—H⋯*A*	*D*—H	H⋯*A*	*D*⋯*A*	*D*—H⋯*A*
C7—H7*A*⋯O1^i^	0.95	2.40	3.213 (3)	143
C29—H29*A*⋯F2^ii^	0.98	2.54	3.447 (3)	155
C29—H29*B*⋯*Cg*1^iii^	0.98	2.75	3.521 (3)	136

Symmetry codes: (i) 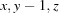; (ii) 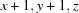; (iii) 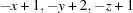.

**Fig. 1.**
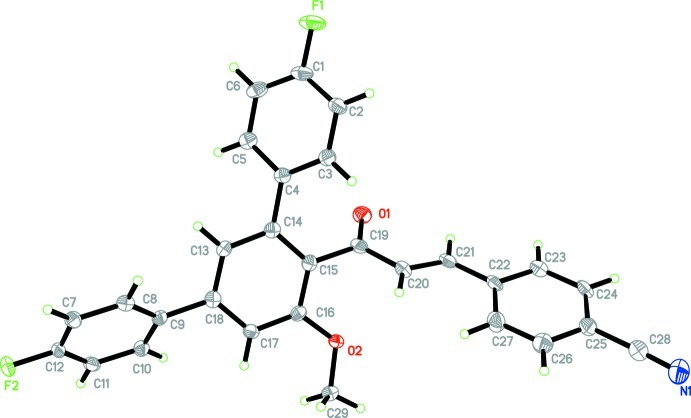
The molecular structure of the title compound, showing 50% probability displacement ellipsoids.

**Fig. 2.**
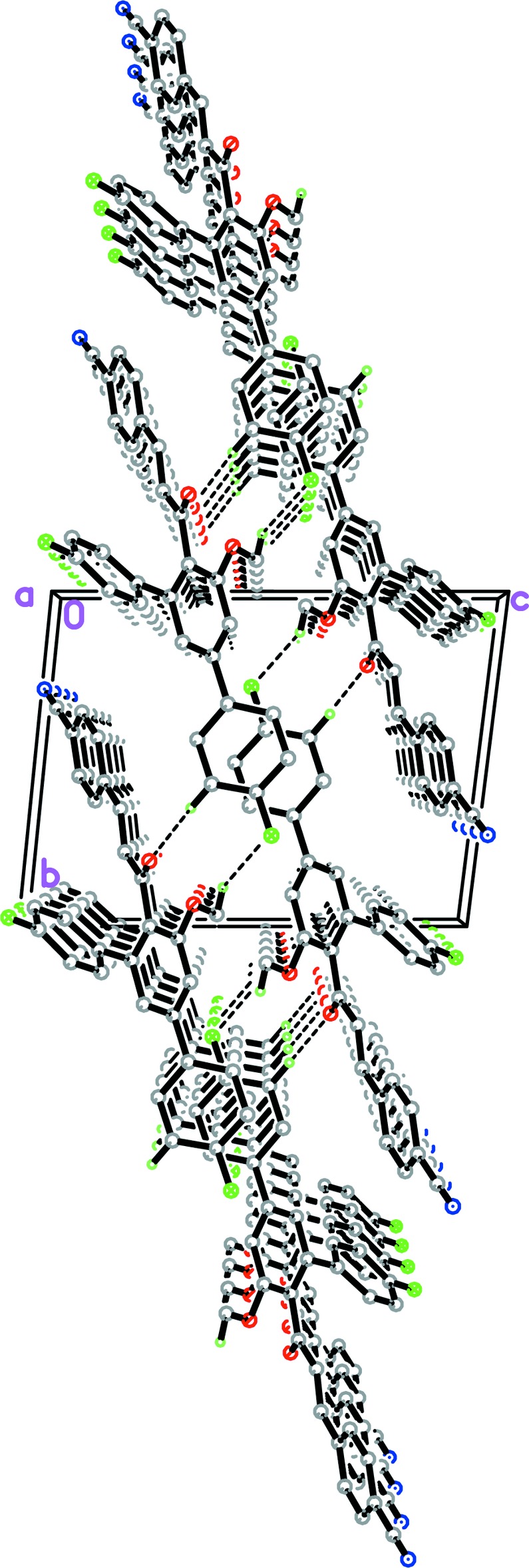
The crystal packing of the title compound, viewed along the a axis, showing the plane parallel to the ab plane. H atoms not involved in the intermolecular interactions (dashed lines) have been omitted for clarity.

## supplementary crystallographic information

### Comment

In continuation of our work on synthesis of terphenyl chalcones (Fun, Hemamalini *et al.*, 2011; Fun, Shahani *et al.*, 2011; Betz *et al.*, 2011), the title compound is prepared and its crystal structure is reported. The starting material of the title compound was prepared from 4,4'-difluoro chalcone by several steps (Fun *et al.*, 2012).

In the title compound (Fig. 1), the central benzene ring (C13–C18) forms dihedral angles of 56.92 (12)° with the cyanobenzene ring (C22–C28/N1) and 40.91 (12) and 44.76 (12)°, respectively, with the fluorobenzene rings (C1–C6/F1 & C7–C12/F2). Bond lengths are angles are within the normal ranges and are comparable with the related structure (Fun, Chia *et al.*, 2011).

In the crystal packing (Fig. 2), intermolecular C7—H7A···O1 and C29—H29A···F2 hydrogen bonds (Table 1) link the molecules to form planes parallel to the *ab* plane. The crystal packing is further stabilized by C—H···π interactions (Table 1), involving the central benzene ring.

### Experimental

To a mixture of 1-(4,4''-difluoro-5'-methoxy-1,1':3',1''-terphenyl-4'-yl) ethanone (0.338 g, 0.001 mol) and 4-cyanobenzaldehyde (0.131 g, 0.001 mol) in 30 ml e thanol, 0.5 ml of 10% sodium hydroxide solution was added and stirred at 5–10°C for 3 h. The precipitate formed was collected by filtration and purified by recrystallization from ethanol. Colourless plates were grown from acetone solution by slow evaporation method and yield of the compound was 78%. *M.p.*: 450 K.

### Refinement

All the H atoms were positioned geometrically and were refined with a riding model with *U*_iso_(H) = 1.2 or 1.5 *U*_eq_(C) [C–H = 0.95 or 0.98 Å]. A rotating group model was applied to the methyl group. In the final refinement, nine outliners were omitted, -6 10 5, 0 0 1, -6 1 0, -5 10 7, -5 11 5, -2 11 8, -6 2 0, -4 9 10 and -2 9 11.

### Crystal data

**Table:**

C_29_H_19_F_2_NO_2_	*Z* = 2
*M**_r_* = 451.45	*F*(000) = 468
Triclinic, *P*1	*D*_x_ = 1.365 Mg m^−^^3^
Hall symbol: -P 1	Mo *K*α radiation, λ = 0.71073 Å
*a* = 6.9656 (2) Å	Cell parameters from 6297 reflections
*b* = 11.2404 (3) Å	θ = 2.2–30.1°
*c* = 14.6014 (3) Å	µ = 0.10 mm^−^^1^
α = 96.108 (1)°	*T* = 100 K
β = 90.415 (1)°	Plate, colourless
γ = 104.764 (1)°	0.34 × 0.20 × 0.12 mm
*V* = 1098.51 (5) Å^3^	

### Data collection

**Table:**

Bruker APEXII CCD diffractometer	3828 independent reflections
Radiation source: fine-focus sealed tube	2982 reflections with *I* > 2σ(*I*)
Graphite monochromator	*R*_int_ = 0.036
φ and ω scans	θ_max_ = 25.0°, θ_min_ = 2.2°
Absorption correction: multi-scan (*SADABS*; Bruker, 2009)	*h* = −8→8
*T*_min_ = 0.968, *T*_max_ = 0.989	*k* = −13→13
17054 measured reflections	*l* = −17→17

### Refinement

**Table:**

Refinement on *F*^2^	Primary atom site location: structure-invariant direct methods
Least-squares matrix: full	Secondary atom site location: difference Fourier map
*R*[*F*^2^ > 2σ(*F*^2^)] = 0.050	Hydrogen site location: inferred from neighbouring sites
*wR*(*F*^2^) = 0.112	H-atom parameters constrained
*S* = 1.03	*w* = 1/[σ^2^(*F*_o_^2^) + (0.0128*P*)^2^ + 2.0889*P*] where *P* = (*F*_o_^2^ + 2*F*_c_^2^)/3
3828 reflections	(Δ/σ)_max_ < 0.001
308 parameters	Δρ_max_ = 0.31 e Å^−^^3^
0 restraints	Δρ_min_ = −0.23 e Å^−^^3^

### Special details

**Table:**

Experimental. The crystal was placed in the cold stream of an Oxford Cryosystems Cobra open-flow nitrogen cryostat (Cosier & Glazer, 1986) operating at 100.0 (1) K.
Geometry. All e.s.d.'s (except the e.s.d. in the dihedral angle between two l.s. planes) are estimated using the full covariance matrix. The cell e.s.d.'s are taken into account individually in the estimation of e.s.d.'s in distances, angles and torsion angles; correlations between e.s.d.'s in cell parameters are only used when they are defined by crystal symmetry. An approximate (isotropic) treatment of cell e.s.d.'s is used for estimating e.s.d.'s involving l.s. planes.
Refinement. Refinement of *F*^2^ against ALL reflections. The weighted *R*-factor *wR* and goodness of fit *S* are based on *F*^2^, conventional *R*-factors *R* are based on *F*, with *F* set to zero for negative *F*^2^. The threshold expression of *F*^2^ > σ(*F*^2^) is used only for calculating *R*-factors(gt) *etc*. and is not relevant to the choice of reflections for refinement. *R*-factors based on *F*^2^ are statistically about twice as large as those based on *F*, and *R*- factors based on ALL data will be even larger.

### Fractional atomic coordinates and isotropic or equivalent isotropic displacement parameters (Å^2^)

**Table:**

	*x*	*y*	*z*	*U*_iso_*/*U*_eq_	
F1	−0.3094 (2)	1.07639 (17)	0.96874 (12)	0.0383 (5)	
F2	0.1740 (2)	0.29331 (13)	0.46224 (11)	0.0282 (4)	
O1	0.2862 (3)	1.22148 (17)	0.70626 (13)	0.0252 (4)	
O2	0.6812 (3)	1.09304 (16)	0.60777 (12)	0.0209 (4)	
N1	1.5517 (4)	1.7077 (3)	0.98443 (19)	0.0430 (7)	
C1	−0.1786 (4)	1.0421 (3)	0.90947 (19)	0.0256 (6)	
C2	0.0215 (4)	1.0968 (3)	0.92571 (19)	0.0235 (6)	
H2A	0.0668	1.1560	0.9778	0.028*	
C3	0.1545 (4)	1.0639 (2)	0.86487 (18)	0.0204 (6)	
H3A	0.2927	1.1003	0.8758	0.025*	
C4	0.0899 (4)	0.9781 (2)	0.78753 (17)	0.0181 (6)	
C5	−0.1138 (4)	0.9222 (3)	0.77510 (18)	0.0216 (6)	
H5A	−0.1603	0.8618	0.7239	0.026*	
C6	−0.2491 (4)	0.9536 (3)	0.83625 (19)	0.0251 (6)	
H6A	−0.3871	0.9148	0.8278	0.030*	
C7	0.2004 (4)	0.4356 (2)	0.59530 (19)	0.0225 (6)	
H7A	0.1618	0.3693	0.6322	0.027*	
C8	0.2424 (4)	0.5575 (2)	0.63458 (18)	0.0204 (6)	
H8A	0.2313	0.5749	0.6992	0.025*	
C9	0.3007 (4)	0.6554 (2)	0.58073 (18)	0.0185 (6)	
C10	0.3164 (4)	0.6277 (2)	0.48615 (18)	0.0205 (6)	
H10A	0.3577	0.6931	0.4487	0.025*	
C11	0.2725 (4)	0.5059 (2)	0.44581 (18)	0.0210 (6)	
H11A	0.2813	0.4872	0.3812	0.025*	
C12	0.2160 (4)	0.4130 (2)	0.50176 (19)	0.0206 (6)	
C13	0.2098 (4)	0.8217 (2)	0.68521 (17)	0.0183 (6)	
H13A	0.1030	0.7600	0.7052	0.022*	
C14	0.2329 (4)	0.9455 (2)	0.72000 (17)	0.0179 (6)	
C15	0.3935 (4)	1.0359 (2)	0.69134 (17)	0.0181 (6)	
C16	0.5288 (4)	0.9990 (2)	0.63103 (17)	0.0181 (6)	
C17	0.5009 (4)	0.8762 (2)	0.59581 (17)	0.0182 (6)	
H17A	0.5919	0.8531	0.5537	0.022*	
C18	0.3390 (4)	0.7864 (2)	0.62211 (17)	0.0188 (6)	
C19	0.4204 (4)	1.1726 (2)	0.71882 (17)	0.0198 (6)	
C20	0.6148 (4)	1.2424 (2)	0.76212 (17)	0.0214 (6)	
H20A	0.7079	1.1977	0.7764	0.026*	
C21	0.6651 (4)	1.3647 (2)	0.78190 (18)	0.0216 (6)	
H21A	0.5699	1.4077	0.7672	0.026*	
C22	0.8547 (4)	1.4389 (2)	0.82446 (18)	0.0216 (6)	
C23	0.8852 (4)	1.5658 (3)	0.84806 (19)	0.0274 (7)	
H23A	0.7825	1.6040	0.8354	0.033*	
C24	1.0628 (4)	1.6374 (3)	0.8896 (2)	0.0292 (7)	
H24A	1.0814	1.7239	0.9054	0.035*	
C25	1.2137 (4)	1.5813 (3)	0.90810 (18)	0.0269 (7)	
C26	1.1865 (4)	1.4552 (3)	0.8842 (2)	0.0295 (7)	
H26A	1.2891	1.4170	0.8971	0.035*	
C27	1.0109 (4)	1.3854 (3)	0.8418 (2)	0.0282 (7)	
H27A	0.9950	1.2996	0.8239	0.034*	
C28	1.4004 (5)	1.6533 (3)	0.9511 (2)	0.0325 (7)	
C29	0.8394 (4)	1.0612 (3)	0.55730 (19)	0.0234 (6)	
H29A	0.9443	1.1364	0.5510	0.035*	
H29B	0.7889	1.0205	0.4960	0.035*	
H29C	0.8936	1.0048	0.5903	0.035*	

### Atomic displacement parameters (Å^2^)

**Table:**

	*U*^11^	*U*^22^	*U*^33^	*U*^12^	*U*^13^	*U*^23^
F1	0.0321 (10)	0.0470 (12)	0.0418 (11)	0.0210 (9)	0.0180 (8)	0.0045 (9)
F2	0.0287 (9)	0.0151 (9)	0.0386 (10)	0.0049 (7)	0.0016 (7)	−0.0046 (7)
O1	0.0235 (10)	0.0234 (11)	0.0322 (11)	0.0118 (9)	0.0014 (8)	0.0047 (9)
O2	0.0191 (9)	0.0167 (10)	0.0261 (10)	0.0039 (8)	0.0083 (8)	−0.0002 (8)
N1	0.0341 (16)	0.0406 (17)	0.0495 (17)	0.0063 (13)	−0.0063 (13)	−0.0082 (14)
C1	0.0269 (15)	0.0285 (17)	0.0281 (16)	0.0161 (13)	0.0122 (12)	0.0100 (13)
C2	0.0282 (15)	0.0214 (15)	0.0230 (14)	0.0103 (12)	0.0066 (12)	0.0019 (12)
C3	0.0202 (14)	0.0192 (15)	0.0232 (14)	0.0065 (11)	0.0020 (11)	0.0044 (11)
C4	0.0190 (13)	0.0175 (14)	0.0204 (14)	0.0079 (11)	0.0023 (11)	0.0059 (11)
C5	0.0200 (14)	0.0239 (16)	0.0230 (14)	0.0078 (12)	−0.0001 (11)	0.0061 (12)
C6	0.0168 (14)	0.0296 (17)	0.0334 (16)	0.0106 (12)	0.0042 (12)	0.0124 (13)
C7	0.0206 (14)	0.0173 (15)	0.0319 (16)	0.0076 (11)	0.0036 (12)	0.0056 (12)
C8	0.0197 (14)	0.0224 (15)	0.0199 (14)	0.0067 (12)	0.0025 (11)	0.0020 (11)
C9	0.0124 (12)	0.0184 (15)	0.0241 (14)	0.0032 (11)	−0.0006 (10)	0.0014 (11)
C10	0.0186 (13)	0.0183 (15)	0.0246 (14)	0.0047 (11)	0.0007 (11)	0.0028 (12)
C11	0.0178 (14)	0.0231 (15)	0.0218 (14)	0.0069 (11)	0.0019 (11)	−0.0025 (12)
C12	0.0142 (13)	0.0150 (14)	0.0316 (16)	0.0045 (11)	0.0000 (11)	−0.0036 (12)
C13	0.0160 (13)	0.0183 (15)	0.0195 (13)	0.0023 (11)	0.0000 (11)	0.0030 (11)
C14	0.0155 (13)	0.0217 (15)	0.0169 (13)	0.0057 (11)	−0.0032 (10)	0.0025 (11)
C15	0.0174 (13)	0.0193 (15)	0.0175 (13)	0.0043 (11)	−0.0025 (11)	0.0023 (11)
C16	0.0149 (13)	0.0209 (15)	0.0184 (13)	0.0039 (11)	−0.0013 (11)	0.0031 (11)
C17	0.0171 (13)	0.0205 (15)	0.0175 (13)	0.0064 (11)	0.0008 (10)	0.0003 (11)
C18	0.0189 (13)	0.0193 (15)	0.0188 (13)	0.0060 (11)	−0.0029 (11)	0.0034 (11)
C19	0.0243 (14)	0.0209 (15)	0.0161 (13)	0.0087 (12)	0.0051 (11)	0.0027 (11)
C20	0.0257 (15)	0.0197 (16)	0.0204 (14)	0.0096 (12)	0.0013 (11)	−0.0004 (11)
C21	0.0245 (14)	0.0210 (16)	0.0214 (14)	0.0108 (12)	0.0062 (11)	−0.0005 (12)
C22	0.0278 (15)	0.0187 (15)	0.0187 (14)	0.0085 (12)	0.0067 (11)	−0.0024 (11)
C23	0.0279 (16)	0.0258 (17)	0.0288 (16)	0.0097 (13)	0.0046 (12)	−0.0029 (13)
C24	0.0333 (17)	0.0211 (16)	0.0304 (16)	0.0072 (13)	0.0086 (13)	−0.0097 (13)
C25	0.0271 (15)	0.0295 (17)	0.0204 (14)	0.0034 (13)	0.0034 (12)	−0.0039 (12)
C26	0.0292 (16)	0.0287 (17)	0.0312 (16)	0.0088 (13)	0.0007 (13)	0.0024 (13)
C27	0.0312 (16)	0.0199 (15)	0.0326 (16)	0.0057 (13)	−0.0002 (13)	0.0016 (13)
C28	0.0315 (17)	0.0304 (18)	0.0329 (17)	0.0068 (14)	0.0027 (14)	−0.0051 (14)
C29	0.0192 (14)	0.0241 (16)	0.0265 (15)	0.0047 (12)	0.0046 (11)	0.0026 (12)

### Geometric parameters (Å, º)

**Table:**

F1—C1	1.360 (3)	C13—C18	1.390 (4)
F2—C12	1.364 (3)	C13—C14	1.398 (4)
O1—C19	1.221 (3)	C13—H13A	0.9500
O2—C16	1.367 (3)	C14—C15	1.406 (4)
O2—C29	1.429 (3)	C15—C16	1.403 (4)
N1—C28	1.150 (4)	C15—C19	1.510 (4)
C1—C6	1.375 (4)	C16—C17	1.385 (4)
C1—C2	1.380 (4)	C17—C18	1.394 (4)
C2—C3	1.379 (4)	C17—H17A	0.9500
C2—H2A	0.9500	C19—C20	1.479 (4)
C3—C4	1.396 (4)	C20—C21	1.328 (4)
C3—H3A	0.9500	C20—H20A	0.9500
C4—C5	1.400 (4)	C21—C22	1.464 (4)
C4—C14	1.490 (4)	C21—H21A	0.9500
C5—C6	1.387 (4)	C22—C23	1.392 (4)
C5—H5A	0.9500	C22—C27	1.404 (4)
C6—H6A	0.9500	C23—C24	1.387 (4)
C7—C12	1.373 (4)	C23—H23A	0.9500
C7—C8	1.385 (4)	C24—C25	1.395 (4)
C7—H7A	0.9500	C24—H24A	0.9500
C8—C9	1.398 (4)	C25—C26	1.387 (4)
C8—H8A	0.9500	C25—C28	1.443 (4)
C9—C10	1.394 (4)	C26—C27	1.376 (4)
C9—C18	1.488 (4)	C26—H26A	0.9500
C10—C11	1.388 (4)	C27—H27A	0.9500
C10—H10A	0.9500	C29—H29A	0.9800
C11—C12	1.374 (4)	C29—H29B	0.9800
C11—H11A	0.9500	C29—H29C	0.9800
			
C16—O2—C29	118.1 (2)	C14—C15—C19	122.5 (2)
F1—C1—C6	119.2 (2)	O2—C16—C17	123.8 (2)
F1—C1—C2	118.5 (3)	O2—C16—C15	115.1 (2)
C6—C1—C2	122.3 (3)	C17—C16—C15	121.1 (2)
C3—C2—C1	118.7 (3)	C16—C17—C18	120.0 (2)
C3—C2—H2A	120.7	C16—C17—H17A	120.0
C1—C2—H2A	120.7	C18—C17—H17A	120.0
C2—C3—C4	121.2 (2)	C13—C18—C17	119.1 (2)
C2—C3—H3A	119.4	C13—C18—C9	120.3 (2)
C4—C3—H3A	119.4	C17—C18—C9	120.5 (2)
C3—C4—C5	118.2 (2)	O1—C19—C20	122.5 (2)
C3—C4—C14	121.3 (2)	O1—C19—C15	120.8 (2)
C5—C4—C14	120.5 (2)	C20—C19—C15	116.7 (2)
C6—C5—C4	121.2 (3)	C21—C20—C19	123.0 (3)
C6—C5—H5A	119.4	C21—C20—H20A	118.5
C4—C5—H5A	119.4	C19—C20—H20A	118.5
C1—C6—C5	118.4 (3)	C20—C21—C22	125.6 (3)
C1—C6—H6A	120.8	C20—C21—H21A	117.2
C5—C6—H6A	120.8	C22—C21—H21A	117.2
C12—C7—C8	118.3 (3)	C23—C22—C27	118.2 (3)
C12—C7—H7A	120.9	C23—C22—C21	120.4 (2)
C8—C7—H7A	120.9	C27—C22—C21	121.5 (2)
C7—C8—C9	121.1 (2)	C24—C23—C22	121.3 (3)
C7—C8—H8A	119.5	C24—C23—H23A	119.4
C9—C8—H8A	119.5	C22—C23—H23A	119.4
C10—C9—C8	118.5 (2)	C23—C24—C25	119.4 (3)
C10—C9—C18	120.2 (2)	C23—C24—H24A	120.3
C8—C9—C18	121.2 (2)	C25—C24—H24A	120.3
C11—C10—C9	121.0 (3)	C26—C25—C24	120.1 (3)
C11—C10—H10A	119.5	C26—C25—C28	119.2 (3)
C9—C10—H10A	119.5	C24—C25—C28	120.8 (3)
C12—C11—C10	118.3 (2)	C27—C26—C25	120.0 (3)
C12—C11—H11A	120.9	C27—C26—H26A	120.0
C10—C11—H11A	120.9	C25—C26—H26A	120.0
F2—C12—C7	118.8 (2)	C26—C27—C22	121.0 (3)
F2—C12—C11	118.3 (2)	C26—C27—H27A	119.5
C7—C12—C11	122.9 (2)	C22—C27—H27A	119.5
C18—C13—C14	121.7 (2)	N1—C28—C25	177.9 (3)
C18—C13—H13A	119.1	O2—C29—H29A	109.5
C14—C13—H13A	119.1	O2—C29—H29B	109.5
C13—C14—C15	118.8 (2)	H29A—C29—H29B	109.5
C13—C14—C4	119.5 (2)	O2—C29—H29C	109.5
C15—C14—C4	121.7 (2)	H29A—C29—H29C	109.5
C16—C15—C14	119.2 (2)	H29B—C29—H29C	109.5
C16—C15—C19	118.3 (2)		
			
F1—C1—C2—C3	−178.9 (2)	C19—C15—C16—O2	4.0 (3)
C6—C1—C2—C3	1.8 (4)	C14—C15—C16—C17	3.3 (4)
C1—C2—C3—C4	0.7 (4)	C19—C15—C16—C17	−173.4 (2)
C2—C3—C4—C5	−2.5 (4)	O2—C16—C17—C18	−178.8 (2)
C2—C3—C4—C14	177.9 (2)	C15—C16—C17—C18	−1.7 (4)
C3—C4—C5—C6	1.8 (4)	C14—C13—C18—C17	2.8 (4)
C14—C4—C5—C6	−178.6 (2)	C14—C13—C18—C9	−175.0 (2)
F1—C1—C6—C5	178.3 (2)	C16—C17—C18—C13	−1.3 (4)
C2—C1—C6—C5	−2.5 (4)	C16—C17—C18—C9	176.5 (2)
C4—C5—C6—C1	0.6 (4)	C10—C9—C18—C13	134.0 (3)
C12—C7—C8—C9	−0.5 (4)	C8—C9—C18—C13	−43.9 (4)
C7—C8—C9—C10	−0.1 (4)	C10—C9—C18—C17	−43.7 (3)
C7—C8—C9—C18	177.8 (2)	C8—C9—C18—C17	138.3 (3)
C8—C9—C10—C11	0.9 (4)	C16—C15—C19—O1	124.0 (3)
C18—C9—C10—C11	−177.1 (2)	C14—C15—C19—O1	−52.6 (4)
C9—C10—C11—C12	−1.0 (4)	C16—C15—C19—C20	−56.7 (3)
C8—C7—C12—F2	−179.7 (2)	C14—C15—C19—C20	126.7 (3)
C8—C7—C12—C11	0.5 (4)	O1—C19—C20—C21	−7.0 (4)
C10—C11—C12—F2	−179.6 (2)	C15—C19—C20—C21	173.7 (2)
C10—C11—C12—C7	0.3 (4)	C19—C20—C21—C22	−179.9 (2)
C18—C13—C14—C15	−1.2 (4)	C20—C21—C22—C23	−175.3 (3)
C18—C13—C14—C4	180.0 (2)	C20—C21—C22—C27	5.3 (4)
C3—C4—C14—C13	137.4 (3)	C27—C22—C23—C24	−1.6 (4)
C5—C4—C14—C13	−42.2 (3)	C21—C22—C23—C24	179.1 (3)
C3—C4—C14—C15	−41.4 (4)	C22—C23—C24—C25	0.0 (4)
C5—C4—C14—C15	139.0 (3)	C23—C24—C25—C26	0.6 (4)
C13—C14—C15—C16	−1.9 (4)	C23—C24—C25—C28	179.8 (3)
C4—C14—C15—C16	176.9 (2)	C24—C25—C26—C27	0.4 (4)
C13—C14—C15—C19	174.7 (2)	C28—C25—C26—C27	−178.9 (3)
C4—C14—C15—C19	−6.5 (4)	C25—C26—C27—C22	−2.0 (4)
C29—O2—C16—C17	−11.4 (3)	C23—C22—C27—C26	2.5 (4)
C29—O2—C16—C15	171.4 (2)	C21—C22—C27—C26	−178.1 (3)
C14—C15—C16—O2	−179.3 (2)		

### Hydrogen-bond geometry (Å, º)

Cg1 is the centroid of the C13–C18 ring.

**Table:**

*D*—H···*A*	*D*—H	H···*A*	*D*···*A*	*D*—H···*A*
C7—H7*A*···O1^i^	0.95	2.40	3.213 (3)	143
C29—H29*A*···F2^ii^	0.98	2.54	3.447 (3)	155
C29—H29*B*···*Cg*1^iii^	0.98	2.75	3.521 (3)	136

Symmetry codes: (i) *x*, *y*−1, *z*; (ii) *x*+1, *y*+1, *z*; (iii) −*x*+1, −*y*+2, −*z*+1.
